# Complementary mechanisms for neurotoxin resistance in a copepod

**DOI:** 10.1038/s41598-017-14545-z

**Published:** 2017-10-27

**Authors:** Vittoria Roncalli, Petra H. Lenz, Matthew C. Cieslak, Daniel K. Hartline

**Affiliations:** Békésy Laboratory of Neurobiology, Pacific Biosciences Research Center, School of Ocean and Earth Science and Technology, University of Hawai’i at Manoa, 1993 East-West Road, Honolulu, HI 96822 USA

## Abstract

Toxin resistance is a recurring evolutionary response by predators feeding on toxic prey. These adaptations impact physiological interaction and community ecology. Mechanisms for resistance vary depending on the predator and the nature of the toxin. Potent neurotoxins like tetrodotoxin (TTX) and saxitoxin (STX) that are highly toxic to humans and other vertebrates, target conserved voltage-gated sodium channels (Na_V_) of nerve and muscle, causing paralysis. The copepod *Calanus finmarchicus* consumes the STX-producing dinoflagellate, *Alexandrium fundyense* with no effect on survival. Using transcriptomic approaches to search for the mechanism that confers resistance in *C. finmarchicus*, we identified splice variants of Na_V_s that were predicted to be toxin resistant. These were co-expressed with putatively non-resistant form in all developmental stages. However its expression was unresponsive to toxin challenge nor was there any up-regulation of genes involved in multi-xenobiotic resistance (MXR) or detoxification (phases I or II). Instead, adults consistently regulated genes encoding digestive enzymes, possibly to complement channel resistance by limiting toxin assimilation via the digestive process. The nauplii, which were more susceptible to STX, did not regulate these enzymes. This study demonstrates how deep-sequencing technology can elucidate multiple mechanisms of toxin resistance concurrently, revealing the linkages between molecular/cellular adaptations and the ecology of an organism.

## Introduction

Production or accumulation of toxins is a common anti-predator measure among plants and animals. In the evolutionary “arms race” between predator and prey, toxic prey is often countered by the evolution of mechanisms that confer toxin resistance to the predator. These mechanisms include mutations in the physiological target that render the toxin less effective^1^, modification of the expression of the target gene/pathway to compensate for its blockage^2^, down-regulation of the target in combination with up-regulation of an alternative toxin-resistant pathway/protein^3^, up-regulation of genes that prevent toxin uptake^2,4^ (e.g. multi-xenobiotic resistance) and up-regulation of biotransformation pathways^4–6^ (e.g. detoxification, phases I and II). The guanidinium alkaloids tetrodotoxin (TTX) and saxitoxin (STX) are potent anti-predator neurotoxins characterized as “keystone molecules” owing to their presence in prey organisms impacting a broad spectrum of predators and ecosystems, including humans, other vertebrates, and many invertebrates^7^. Cases of TTX and STX-resistant organisms have been found, among garter snakes (*Thamnophis* spp.)^8^, shellfish (*Mya arenaria*, *Saxidomus giganteus*)^9^, puffer fish (*Tetraodon nigroviridis*)^10^ and copepods (*Calanus finmarchicus, Acartia hudsonica*)^11,12^. STX and TTX act by binding to voltage-gated sodium channels, blocking sodium-dependent action potentials of nerve and (in vertebrates) muscle cells, therefore leading to paralysis and death in non-resistant organisms^13^. A prominent mechanism that confers resistance is the presence of mutations in the channel protein that decrease toxin binding^1,10,14–17^.

In marine ecosystems, the primary source of STX is from harmful algal blooms, also known as “red tides”, caused by a variety of toxin-producing dinoflagellates in the genus *Alexandrium* ^18^. Red tides, which are responsible for outbreaks of paralytic shellfish poisoning, occur worldwide and have major ecological and economic impact in coastal regions along both Atlantic and Pacific coasts of North America as far north as the Arctic Ocean (Chukchi Sea)^18,19^. Annual outbreaks of Paralytic Shellfish Poisoning (PSP) in the Gulf of Maine, are caused by blooms of *Alexandrium fundyense*, which is accumulated in clams and other shellfish^18^. In addition, other planktonic herbivores feed on the dinoflagellate with no ill effect on their survival^20^ suggesting that they might have evolved mechanisms that confer STX resistance. While physiological studies on non-model species can be difficult, transcriptomic approaches hold promise for the assessment of multiple mechanisms by combining an analysis of protein sequences for the presence of mutations with physiological responses at the gene-expression level.

Among the plankton, a major consumer of the dinoflagellate is the filter-feeding copepod *Calanus finmarchicus*, which is one of the more abundant calanoid copepods in the North Atlantic, extending from the mid-Atlantic Shelf off the US east coast to the Barents Sea north of Norway. *C. finmarchicus* serves as major food source for many invertebrates and vertebrates, including whales^21–23^. In studies aimed at identifying the effects of STX ingestion on these organisms, Roncalli and colleagues fed adult females on two doses of toxic *Alexandrium fundyense* for seven days to assess mortality, egg production, egg viability and transcriptomic response^24–26^. Ingestion of the toxic dinoflagellate did not increase mortality in adult *C. finmarchicus*, but had negative effects on reproduction and physiology. Differential gene expression indicated that the copepod responded to the toxic diet with an initial cellular stress response, followed by a metabolic response, indicating that the *A. fundyense* diet was a less-efficient energy source for the copepod^24–27^. However, the basis of STX-resistance in the copepod remained unclear: there was no evidence for the up-regulation of transcripts encoding for the voltage-gated sodium channel, nor was there any evidence for the up-regulation of detoxification pathways, even though these transcripts were well-represented in the reference transcriptome^25–27^. Only 25 genes were consistently regulated at both *A. fundyense* doses and time points, and the majority of these (24) were involved in digestion.

To elucidate the source of STX-resistance of *C. finmarchicus*, we used our previously generated transcriptome^28^ to retrieve sodium channel sequences and search for mutations potentially affecting STX binding. The first transcriptome was generated from individuals from a Gulf of Maine population (GOM), while the second source consisted of individuals from the Norwegian Sea (NOR). In addition, we extended the search for resistance-related changes in gene expression to naupliar stages. While the effect of ingestion of *A. fundyense* on adult *C. finmarchicus* was the focus of the previous study^24–26^, little is known about how the dinoflagellate affects the early developmental stages, which are likely to be more sensitive than older stages, as is the case with *M. arenaria* ^29^. Nauplii grow rapidly through recurring molt-cycles. To test their sensitivity, we fed *C. finmarchicus* late nauplii on a diet of *A. fundyense* for two days, measured their survival, assessed their behavior and quantified relative gene expression. The resulting sensitivity profile was then compared with that reported for adult females to search for mechanisms of resistance^25,27^.

## Results

### Identification of possible STX-resistant Na_V_ channels in *C. finmarchicus*

**Figure 1:**
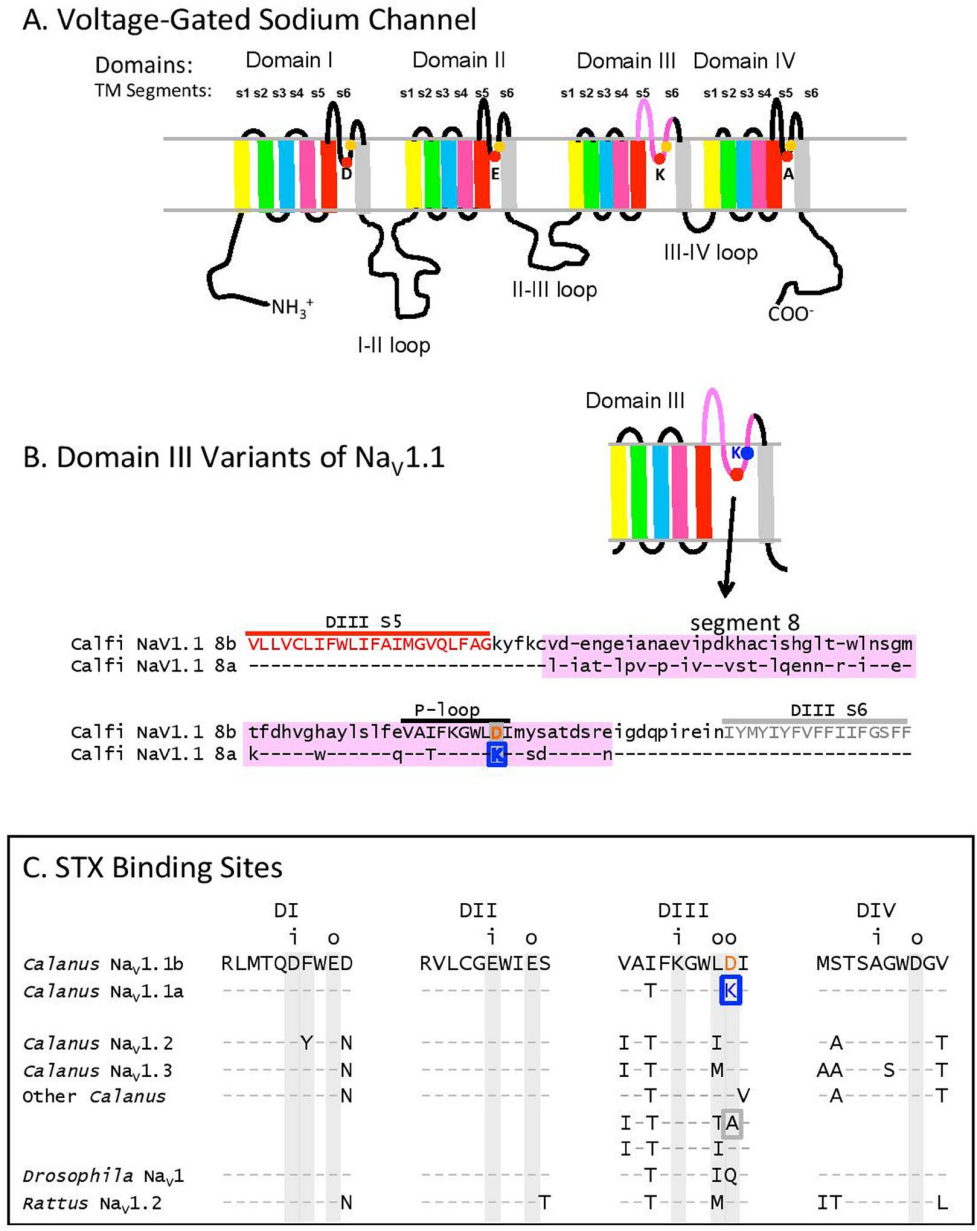
Voltage-gated sodium channel (NaV1). (**A**) Diagram of the channel protein showing the 4 conserved domains (Domains I–IV) with 6 trans-membrane segments each, designated S1–S6, and connected through “linker” sequences. The reentrant “P-loop,” including a selectivity filter residue (red circle) and toxin binding sites (orange and blue circles) enters and exits the confines of the membrane between S5 and S6 of each domain. (**B**) *Domain III variants of Calfi-NaV1.1*.Diagram of Domain III and alignment of the 8a and 8b variant regions (highlighting) of Calfi-NaV1.1, including the P-loops (labeled) flanked by shared common sequence regions of nucleotide segments 7 and 9 (see^28^ for sequence details). Top row shows the amino acid single-letter code for NaV1.1-8b as a reference; Letter codes on the 8a line indicate deviations from the 8b pattern (identical residues indicated by dashes). (**C**) *STX Binding sites*. Sequence alignments for the P-loops of *Calanus finmarchicus*, an insect and a mammal. Only residues deviating from corresponding ones in *Calanus* NaV1.1b (top row) are shown. NaV1.1a is a splice variant of NaV1.1b having a different DIII P-loop sequence. Shading = sites predicted to affect STX binding. Column labels: DI-IV conserved repeat domains; i = inner ring (sodium selectivity filter); o = outer ring; Blue box: toxin binding site with a positive residue (K) replacing a negative one (D); Grey box: a neutral amino acid in the same location. For reference purposes, the inner-ring residues within the NaV1.1-8b sequence are: D376, E1013, K1539, A1832. Accession numbers in Supplementary material, table S1; Calanus NaV1.1a, Calanus NaV1.1b, Calanus NaV1.2, Calanus NaV1.3. Other Accession numbers: *Drosophila melanogaster* sp|P35500; *Rattus norvegicus* sp|P04775; Other *Calanus* - Domain I: GAXK01036301; Domain II: GAXK01012592; Domain III: GAXK01114023; GAXK01009404 and GAXK01063206; Domain IV: GAXK01022998.

We first examined the possibility that *C. finmarchicus* has, within its genetic makeup, voltage-gated sodium channels (Na_V_) that are resistant to STX. The Na_V_ family of eukaryotic proteins contains a pore-forming molecule around 2,000 amino acids long, comprising four highly-conserved homologous domains (DI - DIV), each with six trans-membrane alpha-helical segments (S1–S6). In each domain, ten amino acids in the linker between segments S5 and S6 form a “P-loop,” which lines the outer vestibule of the pore^30^ as diagrammed in Fig. 1A. The basis for STX/TTX susceptibility, as well as for sodium-ion selectivity, resides in two rings of four amino acids each, an inner ring and an outer ring. The P-loops of each of the four domains contributes one residue to each ring. The two rings surround the pore so that a toxin molecule binding to them physically blocks passage of sodium ions through the pore^14^.

Four voltage-gated sodium channel genes have been predicted previously for *C. finmarchicus* from *in silico* searches of the Gulf of Maine (GOM) transcriptome^28^, an unusual number for an invertebrate, which usually have two, designated Na_V_1 and Na_V_2^31^. Three of the copepod channels are full-length members of the Na_V_1 family, and one is an Na_V_2 channel, which is not considered to be a target for blockage by these toxins^30^ (see Supplementary material, Table S1 for accession numbers). The amino-acid sequences for the various Na_V_1 P-loops found in the GOM transcriptome are shown in Fig. 1B. Shading indicates the residues implicated in STX binding for the inner (i) and outer (o) rings, based on extensive studies on other organisms^15,32–34^. In addition to the P-loops from the three putative identified channel genes (which include two splice variants for the P-loop of Domain III of the Na_V_1.1 gene), six additional isolated P-loops were found in short sequences not assembled into full-length proteins, but matching other Na_V_ sequences closely enough to be considered valid Na_V_ P-loops (“Miscellaneous Cf” in Fig. 1B). We also queried the published transcriptome from a Norwegian (NOR) population^35^ and found many of the same P-loop sequences as in the GOM transcriptome (see Supplementary material, Table S1 for corresponding accession numbers). In all, 15 distinct P-loop sequences were identified. Most showed only minor differences among the *C. finmarchicus* Na_V_1s and compared with insect and mammalian P-loops. However, in two of the sequences, significant differences were found in the outer ring of DIII at one of the toxin-binding sites. In the Na_V_1.1 family of transcripts, one of two presumed splice variants, designated Na_V_1.1-8a isoform has a positively-charged lysine (K1545) at this site instead of the negatively-charged aspartic acid (D1543) found in the corresponding position of every other *C. finmarchicus* full-length isoform, including the 8b presumed splice variant form of Na_V_1.1. The alignment of the two Na_V_1.1 isoforms showing the altered residue is presented in Fig. 1C. The exchange of a negative for a positive charge in this position should have a significant effect on the electrostatic forces affecting STX binding. This assumption was confirmed directly in rat Na_V_1.2 by Terlau *et al*.^36^, who showed that the same substitution in the homologous residue, D1426K, substantially reduces toxin binding. One other copepod P-loop has a modified residue in this position, a neutral alanine (A) instead of aspartic acid (D), on a short sequence fragment (Fig. 1B). In addition, this fragment possesses a threonine in an adjacent position that has been identified by Du *et al*.^37^ as conveying TTX-resistance in a broad range of taxa. Thus there is evidence for two isoforms in the *C. finmarchicus* transcriptome that are predicted to be guanidinium-toxin resistant.

### Expression of mutant and non-mutant Na_V_1.1 in *C. finmarchicus*

**Figure 2:**
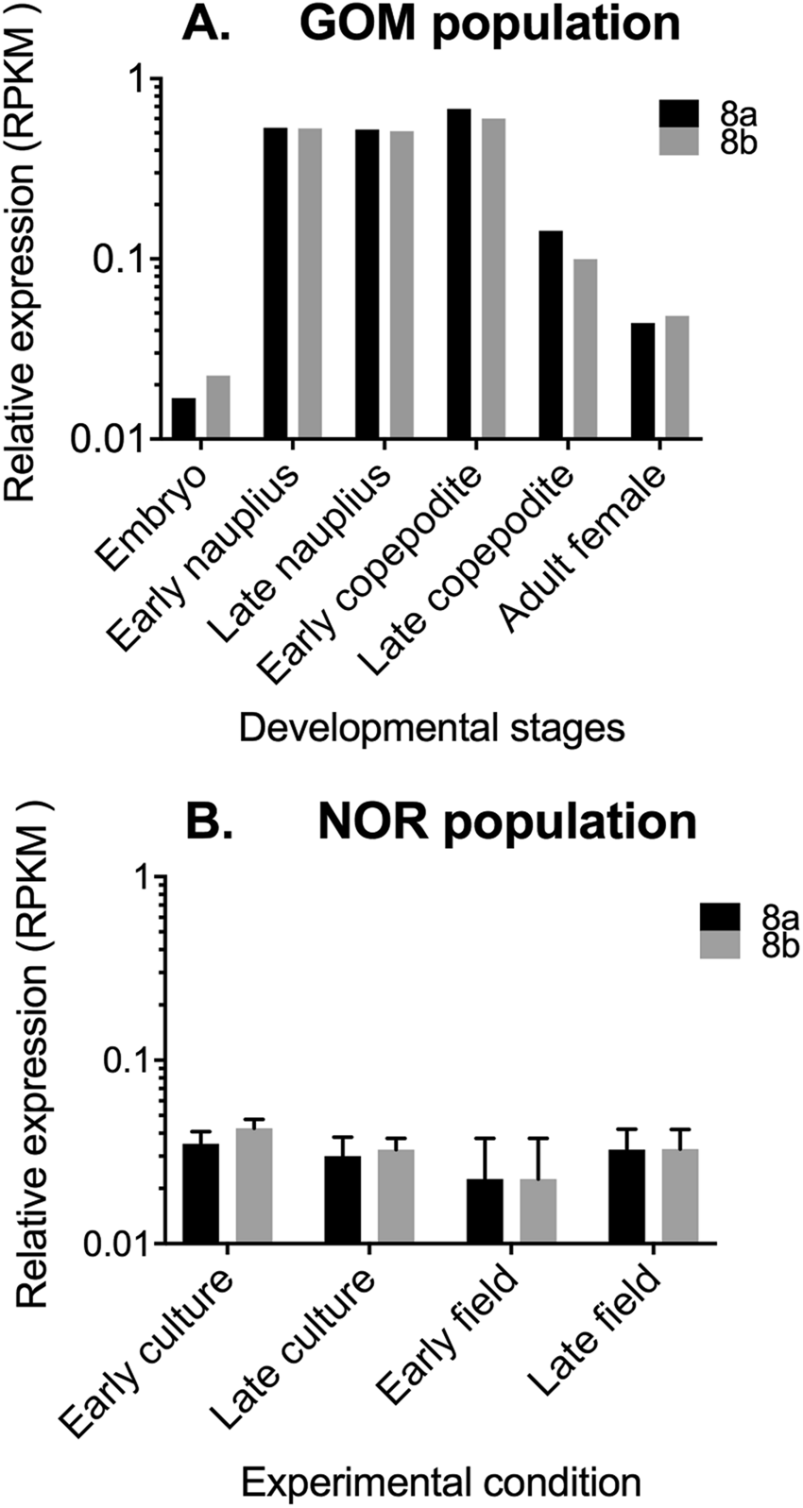
Expression of mutant and non-mutant NaV 1.1 in *C. finmarchicus*. (**A**) Relative expression of segment 8a (black bars) and 8b (grey bars) shown as number of counts per million reads (RPKM) across six developmental stage (embryo, early nauplius, late nauplius, early copepodite, late copepodite, adult female)^28^. (**B**) Relative expression of segment 8a (black bars) and 8b (grey bars) shown as number of counts per million reads (RPKM) in late copepodite (CV) from culture (early and late) and field (early and late)^34^. Error bars shown for the data are standard deviations of four biological replicates. Expression (x-axis) is on a Log10 scale.

Having identified a splice variant in the Na_V_1.1 gene that is predicted, to have reduced STX affinity, targeted mapping was used to examine the relative expression of the two variants, 8a and 8b using publicly available data (NCBI Bioprojects: PRJNA236528 [GOM]: PRJNA231164 [NOR]). In the Gulf of Maine, where *C. finmarchicus* regularly co-occurs with *A. fundyense*, both mutant (8a) and non-mutant (8b) isoforms were expressed in all developmental stages with relative expression of the mutant isoform ranging between 44% and 61% (Fig. 2A), and the proportion of the two isoforms was similar across all stages even though overall expression was not (Fig. 2A). A similar result was obtained for *C. finmarchicus* samples from Norway (Bioproject: PRJNA231164), which originated from both field-collected late copepodites (CV) where *A. fundyense* blooms are rare and from individuals from a long-term continuous culture^35^. The proportion of the mutant isoform (8a) ranged between 43% and 45% for the cultured CVs and between 49% and 53% for field-collected individuals (Fig. 2B).

### Expression of Na_V_ isoforms in *C. finmarchicus* feeding on *A. fundyense*

Relative expression of the Na_V_1.1-8a and 8b isoforms was investigated in *C. finmarchicus* fed on either a control diet or a diet of STX-producing *A. fundyense* using data from the current study and publicly available data (NCBI Bioproject: PRJNA312028 [GOM]). In the previous study, adult females were fed on either a control or two different *A. fundyense* diets (LD: 25% by volume, HD: 100% by volume) for 2 and 5 days^25^. Mapping of reads to either segment was modest ( < 10 mapped reads) and similar in all treatments. No significant differences were observed in overall expression of the Na_V_1.1 gene (or for that matter, Na_V_1.2 or Na_V_1.3) or in the proportion of the two isoforms (GLM test; 2 days: control vs. LD; control vs. HD; 5 days: control vs. LD; control vs. HD; all statistical comparisons: p ≥ 0.90). A similar result was obtained using the targeted mapping of reads to segments 8a and 8b for late nauplii feeding on *A. fundyense* for 2 days (current study). Although relative expression was more than two-fold higher in nauplii than in the adult females, there was no significant difference in expression between treatments (GLM test; control vs. HD: p = 0.9987). Thus, both isoforms are constitutively expressed, and STX exposure did not affect the relative expression of the Na_V_1.1 variants in either adult females or in nauplii. A similar result was found for the expression of the second fragment predicted to be TTX-resistant, with the neutral alanine and adjacent threonine in the outer ring. This, too, did not differ among treatments for adult females or for nauplii. Thus we could detect no transcriptional responsiveness of the voltage-gated sodium channel genes to the toxic alga in either adults or nauplii.

### Effect of *A. fundyense* on late nauplii

#### Feeding, survival and behavior

Evidence for adverse effects of the STX treatments was examined by checking nauplii under a dissecting microscope after 24 and 48-hours for survival, gut fullness and swimming behavior. The guts of the nauplii were colored and filled, indicating that they ingested both the control and experimental algae (*Rhodomonas* sp. and *A. fundyense* respectively). Survival at 48 hours was high with 100% and 95% in the control and experimental treatments (3 replicates per treatment), respectively. However, locomotory activity of nauplii feeding on *A. fundyense* was negatively affected. Control nauplii were actively swimming and producing escape swims, while the nauplii feeding on *A. fundyense* became inactive within 24 hours, lying on the bottom of the container. Movement of their appendages was limited and the nauplii failed to escape from gentle suction. This difference in behavior persisted for the remainder of the experiment (Supplementary material, Table S2).

#### Global gene expression

The transcriptional response observed in nauplii fed on the *A. fundyense* diet involved the differential expression of 814 genes (DEGs), which represented 3% of transcripts expressed at ≥ 1 count per million (cpm). Two thirds (76%) of the DEGs were up-regulated in the experimental nauplii and the remainder down-regulated (Supplementary material, Figure S1, Table S5). Differential gene expression for the majority (90–94%) of up- and down-regulated DEGs was equal to or less than 4-fold (Supplementary material, Figure S1, Table S5).

**Figure 3:**
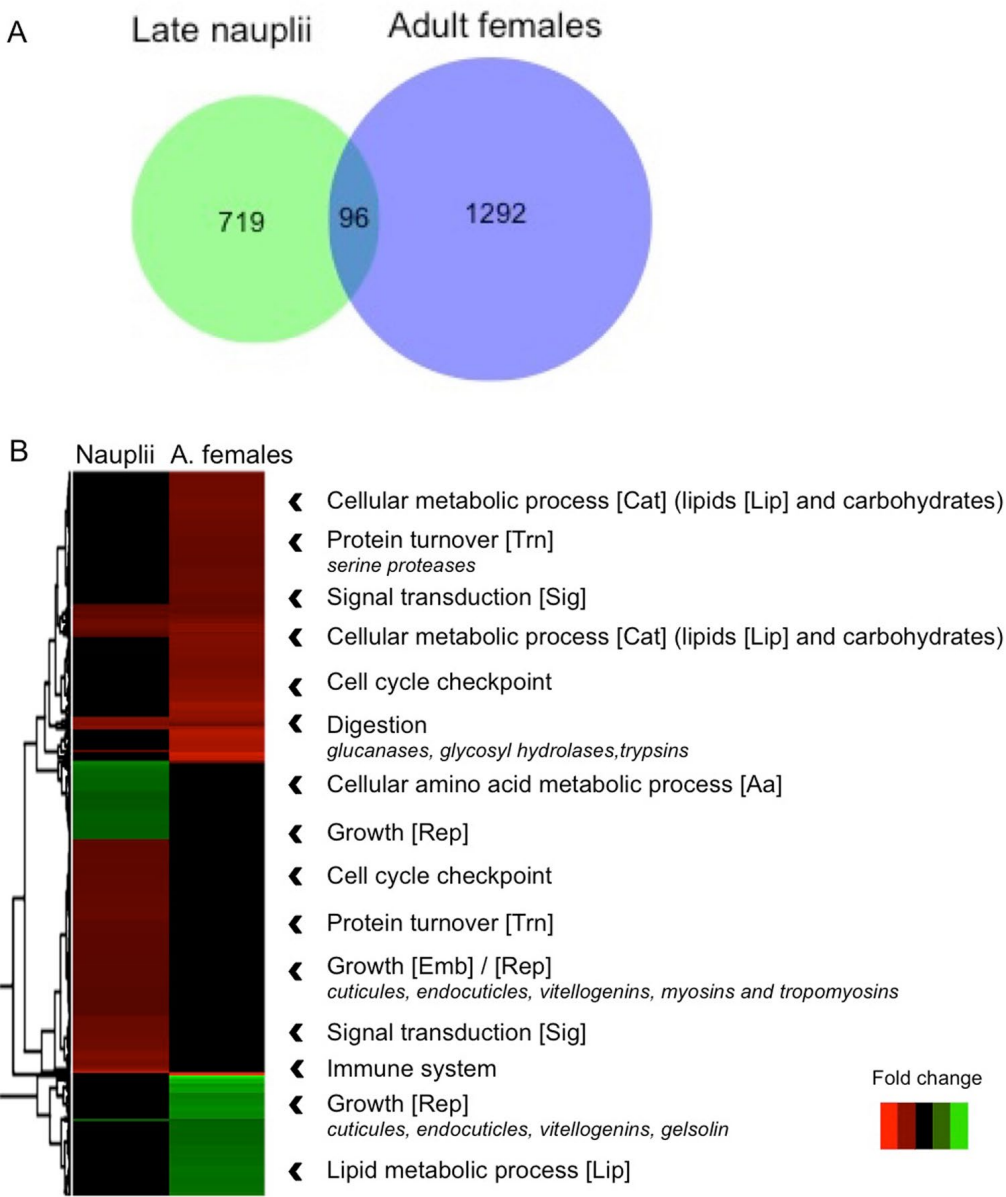
Comparison of *C. finmarchicus* transcriptomic responses between late nauplii and adult females feeding on *A. fundyense* for 2 days. (**A**) Venn diagram of DEGs (up- and down-regulated) in late nauplii and adult females. (**B**) Heat map of annotated DEGs in late nauplii (1st column) and adult females (2nd column). Genes were clustered using *heatmap.2* function (R software) as indicated by the dendrogram on the left side of the figure. Individual biological processes involved in the response are noted on the right side of the figure with the corresponding GO terms in brackets (see Supplemental material, Figure S2). Specific classes of genes are listed (italic) for some of these biological processes as discussed in the text. Relative expression rate (absolute fold change) is calculated for nauplii and adult females feeding on the toxic diet compared with nauplii adult females feeding on the control diet *Rhodomonas* sp. Data for adult females are publicly available through DryAd^2.^

The naupliar response to *A. fundyense* included DEGs from many conserved eukaryotic processes such as cellular metabolic processes, response to stimulus, and growth, (Supplementary material, Figure S2) which included up-regulation for genes involved in signal transduction, protein turnover (transcription), immune system and growth (Fig. 3B). Regulation of these processes is typical for the “cellular stress response” (CSR)^38^, which was confirmed by enrichment analysis of gene ontology (GO) terms: cellular amino acid metabolic process (down-regulated), transport and localization (up-regulated) were all identified as enriched biological processes (Supplementary material, Table S4). Also consistent with the CSR is the up-regulation of genes involved in the degradation of lipids and carbohydrates, and the down-regulation of genes involved in biosynthesis (Fig. 3B).

Enrichment analysis identified the GO term “developmental process” as significantly enriched among the up-regulated transcripts. Regulated genes in this category included several cuticle and endocuticle proteins (flexible, a, 6, 7, 19 classes, number of DEGs = 23), cytoskeletal elements (tubulins, dynein, gelsolin precursor, microtubule associated proteins, number of DEGs = 9) and members of the vitellogenin family (number of DEGs = 10). In addition, several myosins (heavy chain I, II, p and heavy non-muscle), tropomyosins (number of DEGs = 6) and contactins (number of DEGs = 2) were up-regulated. These proteins are not only involved in growth but also in muscle function.

Global gene expression analysis confirmed that none of the Na_V_ transcripts in the reference transcriptome (43 transcripts) were differentially expressed. Furthermore, neither multi-xenobiotic resistance (MXR), nor detoxification was identified as enriched processes among the DEGs. The *C. finmarchicus* reference transcriptome included ca. 50 genes involved in the MXR response and ca. 200 genes involved in either phase I or phase II detoxification^27,28,39^. Searching for these genes among the DEGs in nauplii showed that only 2% of genes involved in these two defense mechanisms (MXR and detoxification, phases I and II) were regulated in response to the dinoflagellate. These DEGs were members of the cytochromes P450 family (phase I detoxification) and all were down-regulated.

### Comparison between transcriptomic responses: adult females vs. nauplii

The naupliar response to *A. fundyense* was compared with the response measured in adult females with the same experimental design and similar algal toxicity (mean 0.02 STX equivalent cell^−1^ d^−1^ ± 0.02 [SD]). Survival of nauplii (95%) and adult females (100%) was high and similar. While the number of DEGs was high in both nauplii (814 DEGs) and females (1388 DEGs), significantly fewer genes were differentially expressed in the nauplii (*X* ^2^ test = 66.02; p < 0.000001; Supplementary material, Table S5) and the number of shared DEGs was small (Fig. 3A).

Both nauplii and adult females responded with a cellular stress response suggesting that the diet is an environmental stressor (Fig. 3B). Up-regulation was observed for genes included in the GO terms “cellular metabolism” (carbohydrate) and “response to stress” (protein turnover, signal transduction, cell cycle checkpoint, immune system) for both nauplii and adult females (Fig. 3B). Seventy % of the 96 DEGs shared between the two stages (Fig. 3A) were involved in “response to stress”; however, even within the CSR, nauplii and adult females regulated different genes (Fig. 3B). For example, within the biological process “protein turnover” members of various classes of serine proteases (3, 6, 9, 14, *easter* and *strubble*; Supplementary material, Table S6) were among the DEGs. Only class 3 was differentially expressed in both; class 9 was specific to the nauplii and the other classes were specific to adult females (Supplementary material, Table S6). Even within class 3, a single member was shared between the stages, while seven additional genes were differentially regulated in the adult females. Expression levels could not explain this difference, since relative expression of serine proteases was similar in the two stages (Supplementary material, Table S6).

The biological process “developmental process” was significantly enriched in nauplii, but not in adult females. The few DEGs involved in growth/reproduction (cuticle and endocuticle proteins [7], vitellogenins [3], gelsolin precursor [1]) that were differentially expressed in females were all down-regulated after two days on the *A. fundyense* diet. In contrast, development and growth transcripts were mostly up-regulated in the nauplii in response to the experimental diet. Relative expression of development and growth genes, including those in the myosin family was higher in the nauplii (RPKM = 1 to 100) than in the females (RPKM = 1 to 60), and some (10%) were silent in the adult females (RPKM = 0). The remaining 67 naupliar DEGs, were expressed at high enough levels in the females and were included in the statistical analysis ( > 1 cpm).

A signature response in the adult females was the regulation of transcripts involved in digestion, and this response was absent in the nauplii. In the adult females, 25 transcripts were differentially expressed under all experimental conditions (LD, HD, 2 days and 5 days)^25,27^. 24 of these DEGs were all associated with digestion and included endoglucanases (7), trypsins (6), glycosil hydrolases (8), lipase (1), phosphogluconolactonase (1) and β-carotene-9-oxygenase (1)^25,27^. These DEGs were up-regulated with the exception of two trypsins that were consistently down-regulated in the females, and the magnitude of the response ranged between 2.5 and 4.3-fold (HD, 2 days). These digestive enzymes were expressed in nauplii at levels between 1 and 71 RPKM, which was similar to the range observed in adult females (RPKM = 1 to 84). In the nauplii modest expression differences (1.6-fold) were observed in six other digestive enzymes (two endoglucanases, two chitinases, one glycosil hydrolase and a carboxyl peptidase).

## Discussion

The results suggest that *C. finmarchicus* STX-resistance is conferred through two complementary mechanisms: 1) an intrinsic STX resistance in certain Na_V_1 variants, co-expressed with non-resistant isoforms but unchanged in expression with *A. fundyense* exposure; and 2) a physiological response that involves the digestive system. While both mechanisms are present in the adult females, the nauplii depend primarily on the first to confer resistance to *A. fundyense*. Nauplii show high survival, but the striking difference in swimming behavior compared with adult females suggests greater susceptibility to STX in the early developmental stages.

### Mechanism 1: Saxitoxin block of voltage-gated sodium channels

#### STX binding to Na_V_s

Within the Na_V_1 P-loops of *C. finmarchicus*, we found isoforms with mutations in two of the nine sites shown in other systems to affect guanidinium ion binding (Fig. 1). One site, D1543 in Calfi Na_V_1.1-8b, corresponds to the residue D1426 in Domain III of the rat brain Na_V_1.2 channel (Fig. 1C), which when mutated from aspartate to lysine (D1426K), reduces STX binding by over 30-fold^36^, a substitution that is the same as in Na_V_1.1 8a. Another Domain III P-loop sequence shown in Fig. 1 (from a partial protein only) had a neutral alanine in this position instead of an aspartate. While Terlau *et al*.^40^ did not test this particular substitution, they did find that substitution of either of two other electrically neutral residues (D1426N and D1426Q) produced a smaller reduction in toxin binding, as might be expected from the lesser charge change. This same sequence also possesses a threonine in an adjacent locus (T1425), where Du *et al*.^37^, observed a pattern of TTX resistance in taxa, ranging from jellyfish to leeches, having this substitution. They followed this up with a study of site-directed mutagenesis on the TTX-resistant mite, *Varroa destructor*. Replacing the threonine with either of two amino acids usually found at that site in non-resistant taxa, as well as *C finmarchicus* (equivalent to T1425I and T1425M), produced a 10-fold greater susceptibility to TTX. The copepod channel with threonine in that locus would thus be expected to show toxin resistance as well.

If indeed Calfi-Na_V_1.1-8a is more STX-resistant than 8b, one might expect that it would be up-regulated in animals on the toxic alga diet. Furthermore, consistent with evidence of higher impairment of nerve and motor function in nauplii, it might be less differentially expressed in those stages, which showed a greater impairment of behavioral responses than did the females. Instead, it appeared that both isoforms were expressed in approximately equal proportions (±50%) in all developmental stages, in individuals from two geographically distant populations (Gulf of Maine and Norway), and in individuals feeding on the toxic dinoflagellate. It might be speculated that with half of the sodium channels protected from the toxin, the loss of function in the remaining channels may not be lethal: there is typically a substantial safety factor for impulse transmission in most nerve. However, if the resistant channel were the only isoform available to the copepod, there could be other negative consequences, as has been found in locomotor deficits in toxin-resistant snakes^8^.

#### Evolution of tolerance to channel-blockers

Genetic modifications become established in a population through natural selection, as has been demonstrated by pesticide-resistance in insects, including that to Na_V_ blockers^41^. Phenotypic variation in the sodium channel is correlated with differences in environmental conditions with toxin-resistant mutants being limited to populations that encounter TTX or STX in their habitat^8^. Garter snakes (*Thamnophis sirtalis*) that co-occur with toxic phenotypes of their salamander prey (*Taricha granulosa*) have evolved novel TTX-resistant Na_V_ mutations independently in several populations^16,34,42^. The toxin-resistance of the snake co-varies with toxin levels in the local prey population^8^. In two resistant snake populations, the aspartic acid (D) of the Domain III (outer ring) is replaced by a glutamic acid (E), but direct evidence that this is the source of TTX-resistance has not been confirmed^34^. For the soft-shell clam, *M. arenaria*, the mutation that confers STX resistance is found in individuals from the Bay of Fundy, known for its harmful algal blooms^15^. Glutamic acid has been replaced by an aspartic acid in the outer ring of the Domain II P-loop (equivalent to E945D in rat Na_V_1.2). In both of these two cases, in contrast to *C. finmarchicus*, there is no charge change at the altered locus. The Domain III mutation in the copepod has not been described occurring naturally in other organisms. However, it is consistent with the convergent evolution of mutations in widely dispersed TTX-resistant snake species and populations with mutations in relatively few of the known possible (TTX/STX)-resistant sites. This has led Feldman and coauthors to suggest that the costs of alternative sites for toxin-resistant mutations may be too high, thus, keeping them from occurring in natural populations^34^.

### Mechanism 2: physiological regulation

Two other toxin-tolerance mechanisms involve physiological regulation by either rendering toxins inactive or limiting their assimilation^6,43,44^.

#### No evidence of activation of two common defense mechanisms: Multi-xenobiotic resistance and detoxification pathways

Up-regulation of enzymes involved in the detoxification pathways (phase I and II) have been reported in the bivalves *Mytilus edulis* and *Crassostrea gigas* and Atlantic salmon (*Salmo salar*) in response to STX-producing *Alexandrium* spp.^45–47^, while STX removal via excretion in *Mytilus galloprovincialis* ^48^ is likely to be mediated by the multi-xenobiotic system^2,48^. In contrast, the response of *C. finmarchicus* to *A. fundyense* did not include either one of two common defense mechanisms: up-regulation of the MXR system or detoxification pathways. While the absence of a detoxification response (phases I and II) has been previously reported in adult females^25,26^, here it is confirmed in late nauplii. Furthermore, there is no evidence for the up-regulation of the first line of defense (MXR) in either females or nauplii, suggesting that STX-tolerance in *C. finmarchicus* occurs via a different mechanism.

#### Role of digestion in detoxification

After ingestion of *A. fundyense*, toxic cells accumulate in the copepod digestive system, where they are broken down prior to absorption. Thus, an alternative mechanism for detoxification would be to limit absorption of the toxin. This mechanism has been suggested as a defense against a toxic *Alexandrium* spp. in the copepod, *Acartia clausi*, and against toxic cyanobacteria in the cladoceran *Daphnia pulex* ^49,50^, albeit based on limited evidence. Other physiological studies on *C. finmarchicus* indicated no difference in respiration rates, but higher levels of digestive enzymes in pre-adult stage CV individuals feeding on toxic *Alexandrium* spp. compared with those on a non-toxic diet^51^. The difference in digestive enzymes is consistent with the transcriptomic response in adult females, which included the up-regulation of multiple genes involved in digestion (24 DEGs). This was the only response that was shared across toxic algal dose and time points out of more than 1,000 DEGs^25^.

Additional support for a defense mechanism that limits absorption of the toxin comes from measurements of STX levels in *C. finmarchicus* ^11,52^. In the clam, *M. arenaria*, which only appears to possess mutation in the Na_V_ channel as mechanism of resistance to STX, retention efficiency in resistant individuals is high and ranges between 60 to 70%^15,29^. This high retention efficiency, as result of the fact that the toxin is not actively removed from the organism, contributes to clams being an important risk factor for human PSP under red tide conditions^15^. In contrast, retention efficiency is low in *C. finmarchicus* and ranges between 2–8% of total ingested toxins^11,52^, which is consistent with a mechanism that limits absorption of the toxin. The mussel *Mytilus edulis*, which also shows low accumulation^53^, eliminates STX presumably through bacterial action in the digestive tract^53^. A detoxification mechanism that involves gut bacteria has been demonstrated in insects^54^ and similar mechanism may be present in the copepods. In the current experiments the females were wild-caught, while the nauplii were laboratory reared, which could have led to differences in their microbiome.

## Methods

### Identification of candidate toxin-resistant Na_V_ channels in *C. finmarchicus*

#### Voltage-gated sodium channel analysis

The voltage-gated sodium channel transcripts identified in the *de novo* assembly of *C. finmarchicus* ^28^ were retrieved and translated using the EMBOSS Transeq web tool^55^ and additional fragments of channels containing P-loops were identified by BLASTing those channel sequences into the transcriptome and checking for a good alignment of the retrieved sequences with the query. Each of the *Calanus* protein fragments was then used as a query in a reciprocal BLAST against the non-redundant proteins curated at NCBI to confirm that the most similar annotated protein in that database was a Na_V_.

#### Relative expression of Na_V_1.1– targeted mapping of reads

We examined the relative expression of the Na_V_1.1-8a and 8b variants (NCBI Acc. No. GAXK01042241) as well as the second fragment predicted to be TTX resistant (NCBI Acc. No. GAXK01009404) in different RNA-Seq *C. finmarchicus* datasets: 1) multiple developmental stages (embryo to adult female) from the Gulf of Maine (GOM) population (NCBI Bioproject PRJNA236528)^28^; 2) late-copepodite (CV) stage individuals from field-collected and cultured Norwegian populations (NCBI Bioproject PRJNA231164)^35^; 3) GOM adult females that had been exposed to three experimental diets (control, low dose *A. fundyense* 25% by volume [LD], and high dose *A. fundyense* 100% by volume [HD])^25^; and 4) late nauplii (NV-NVI) from the GOM population exposed to *A. fundyense* diet (current study; NCBI Bioproject: PRJNA356331).

For each dataset, RNA-Seq reads were mapped against partial sequences of the Na_V_1.1 transcript that included the two variants (8a, 8b) using the software Kallisto (v. 0.43.0), which is designed to accurately map reads to similar sequences^56^; specifically, short Na_V_1.1 reference sequences that included segments “6”, “7” and “8 (a or b)^28^ (see Fig. 1). The lengths of the two alternative reference sequences were 600 and 594 nucleotides for segments 8a and 8b, respectively. The two reference sequences only extended 51 nucleotides beyond segment 8 in order to ensure that the target locus (seg 8) was included in the mapping, while maximizing the number of mapped reads. Similarly, RNA-Seq for each datasets were mapped against the second fragment predicted to be TTX resistant using the software Kallisto (v. 0.43.0)^56^.

Statistical analysis for the relative expression of the Na_V_1.1 gene 8a and 8b isoforms and the TTX-resistant segment across the different datasets was performed using the BioConductor package edgeR using the generalized linear model (GLM) likelihood ratio test with a correction for false discovery using the Benjamini–Hochberg method (false discovery rate [FDR] < 5%)^57^.

### Naupliar response to *A. fundyense*

The experimental design, field collection and cultivation of *C. finmarchicus* as well as RNA-Seq analysis are described in detail in Supplementary material. Briefly, three biological replicates of *C. finmarchicus* “late naupliar stage” (mix of NV and NVI individuals) were incubated at 10 °C and 14 light:10 dark cycle in 100 mL crystallizing dishes with filtered seawater and fed for two days on one of two experimental diets: control and high dose of *A. fundyense* (100% by volume HD) (Supplementary material, Table S2). The control and *A. fundyense* phytoplankton cultures used in this study were the same as those in three parallel studies^24–26^. Nauplii were checked under a dissecting microscope to assess mortality, algal ingestion (colored/filled guts), possible malformations (none were found) and behavior (active swimming, escape swims) after 1 and 2 days. On day 2, nauplii (approximately 70 individuals per sample) were harvested from each treatment and biological replicate and immediately processed for total RNA extraction. cDNA library preparation and high-throughput sequencing was performed for each replicate at the University of Missouri DNA Core Facility (http://biotech.missouri.edu/dnacore). The six libraries were multiplexed and loaded into a single lane and sequenced on an Illumina HiSeq. 2000 instrument using paired-end sequencing (100 bp). Summary of RNA Seq yields are found in Supplementary material, Table S3.

#### Gene expression and functional annotation

Gene expression analysis is described in detail in the Supplementary material. Briefly, quality filtered Illumina reads for the six RNA-Seq libraries were mapped to an existing *C. finmarchicus* reference transcriptome^28^ using software Bowtie (v. 2.0.6)^58^. Relative expression was calculated as reads per kilobase per million mapped reads (RPKM) for each gene using a custom script written in Perl (https://github.com/LenzLab/RNA-seq-scripts). Differential gene expression between the control and experimental treatment (CONTROL vs HD) was calculated using the BioConductor package edgeR^57^ with a TMM normalization (trimmed means of M values) prior the statistical tests. Transcripts with a Benjamini-Hochberg corrected p-value smaller then 0.05 were considered differentially expressed (DEGs). DEGs were annotated against NCBI SwissProt protein database followed by the Gene Ontology (GO) database using UniProt (http://www.uniprot.org/uploadlists/). Enrichment analysis was performed separately for up- and down-regulated genes with GO terms against the genes with assigned GO terms in the *C. finmarchicus* reference transcriptome^27,28^. The analysis was implemented using BLAST2GO (v. 2.6.4) performing the Fisher’s Exact Test followed by Multiple Testing correction of False Discovery rate (FDR < 5%)^59^.

### Comparison with the adult female response to *A. fundyense*

The response to *A. fundyense* measured in the nauplii was compared with the one previously reported in adult females exposed to the same experimental condition^25,27^. The list of DEGs between adult females feeding on *A. fundyense* HD (100% by volume) treatment and a control diet for 2 days included the total number of up- and down-regulated genes (1,388) and the fold change difference in expression between the experimental and control diets^27^. In addition, relative expression levels for different genes were calculated as RPKM as described for the nauplii (RNA-Seq data available on NCBI BioProject: PRJNA312028).

### Data availability

Sequence data have been submitted to the National Center of Biotechnology Information (NCBI; www.ncbi.nlm.nih.gov) under the NCBI Bioproject: PRJNA356331.

## Electronic supplementary material

Supplementary material

## References

[1] McGlothlin JW (2014). Parallel evolution of tetrodotoxin resistance in three voltage-gated sodium channel genes in the garter snake *Thamnophis sirtalis*. Mol. Biol. Evol..

[2] Cree IA, Charlton P (2017). Molecular chess? Hallmarks of anti-cancer drug resistance. BMC cancer.

[3] Tiewsiri K, Wang P (2011). Differential alteration of two aminopeptidases N associated with resistance to *Bacillus thuringiensis* toxin Cry1Ac in cabbage looper. PNAS.

[4] Rushmore TH, Tony Kong A (2002). Pharmacogenomics, regulation and signaling pathways of phase I and II drug metabolizing enzymes. Curr. Drug Metab..

[5] Hemingway J, Ranson H (2000). Insecticide resistance in insect vectors of human disease. Annu. Rev. Entomol..

[6] Rharrabe K, Alla S, Maria A, Sayah F, Lafont R (2007). Diversity of detoxification pathways of ingested ecdysteroids among phytophagous insects. Arch. Insect Biochem. Physiol..

[7] Zimmer RK, Ferrer RP (2007). Neuroecology, chemical defense, and the keystone species concept. Biol. Bull..

[8] Brodie ED (1999). and Brodie Jr. Cost of exploiting poisonous prey: Evolutionary trade-offs in a predator-prey arms race. Evolution.

[9] MacQuarrie SP, Bricelj VM (2008). Behavioral and physiological responses to PSP toxins in *Mya arenaria* populations in relation to previous exposure to red tides. Mar. Ecol. Progr. Ser..

[10] Venkatesh B (2005). Genetic basis of tetrodotoxin resistance in puffer fishes. Curr. Biol..

[11] Teegarden GJ, Cembella AD, Capuano CL, Barron SH, Durbin EG (2003). Phycotoxin accumulation in zooplankton feeding on *Alexandrium fundyense*- vector or sink?. J. Plankton Res..

[12] Colin SP, Dam HG (2002). Latitudinal differentiation in the effects of the toxic dinoflagellate *Alexandrium* spp. on the feeding and reproduction of populations of the copepod *Acartia hudsonica*. Harmful Algae.

[13] Narahashi TO (1972). Mechanism of action of tetrodotoxin and saxitoxin on excitable membranes. Fed. Proc..

[14] Backx PH, Yue DT, Lawrence JH, Marban E, Tomaselli GF (1992). Molecular localization of an ion-binding site within the pore of mammalian sodium channels. Science.

[15] Bricelj VM (2005). Sodium channel mutation leading to saxitoxin resistance in clams increases risk of PSP. Nature.

[16] Geffeney SL, Fujimoto E, Brodie ED, Ruben PC (2005). Evolutionary diversification of TTX-resistant sodium channels in a predator–prey interaction. Nature.

[17] Lee CH, Ruben PC (2008). Interaction between voltage-gated sodium channels and the neurotoxin, tetrodotoxin. Channels.

[18] Rossini, G. P. Toxins And Biologically Active Compounds From Microalgae: Biological Effects And Risk Management. Vol. 2 (CRC Press, 2014).

[19] Natsuike M (2017). Possible spreading of toxic *Alexandrium tamarense* blooms on the Chukchi Sea shelf with the inflow of Pacific summer water due to climatic warming. Harmful Algae.

[20] Turner JT (2014). Planktonic marine copepods and harmful algae. Harmful Algae.

[21] Melle W (2014). The North Atlantic Ocean as habitat for *Calanus finmarchicus*: Environmental factors and life history traits. Progr. Oceanogr..

[22] Darbyson E, Swain DP, Chabot D, Castonguay M (2003). Diel variation in feeding rate and prey composition of herring and mackerel in the southern Gulf of St Lawrence. J. Fish Biol..

[23] Heath MR, Lough RG (2007). A synthesis of large‐scale patterns in the planktonic prey of larval and juvenile cod (*Gadus morhua*). Fish. Oceanogr..

[24] Roncalli V, Turner JT, Kulis D, Anderson DM, Lenz PH (2016). The effect of the toxic dinoflagellate *Alexandrium fundyense* on the  fitness of the calanoid copepod *Calanus finmarchicus*. Harmful Algae.

[25] Roncalli, V., Cieslak, M.C. and Lenz, P.H. Transcriptomic responses of the calanoid copepod *Calanus finmarchicus* to the saxitoxin producing dinoflagellate *Alexandrium fundyense*. *Scientific Reports* **6** (2016). 10.1038/srep25708 PMC4867593 27181871

[26] Roncalli V, Jungbluth MJ, Lenz PH (2016). Glutathione S-Transferase regulation in *Calanus finmarchicus* feeding on the toxic dinoflagellate *Alexandrium fundyense*. PloS one,.

[27] Roncalli, V., Cieslak M.C., Lenz, P. H. Data from: Transcriptomic responses of the calanoid copepod *Calanus finmarchicus* to the saxitoxin producing dinoflagellate *Alexandrium fundyense*. Dryad Digital Repository., 10.5061/dryad.11978 (2016). 10.1038/srep25708 PMC4867593 27181871

[28] Lenz PH (2014). *De novo* assembly of a transcriptome for *Calanus finmarchicus* (Crustacea, Copepoda)–the dominant zooplankter  of the North Atlantic Ocean. PloS one.

[29] Bricelj VM, MacQuarrie SP, Doane JAE, Connell LB (2010). Evidence of selection for resistance to paralytic shellfish toxins during the early life history of soft-shell clam (*Mya arenaria*) populations. Limnol. Oceanogr..

[30] Al-Sabi A, McArthur J, Ostroumov V, French RJ (2006). Marine toxins that target voltage-gated sodium channels. Marine Drugs.

[31] Goldin AL (2002). Evolution of voltage-gated Na+ channels. J Exp Biol.

[32] Lipkind GM, Fozzard HA (1994). A structural model of the tetrodotoxin and saxitoxin binding site of the Na+ channel. Biophys. J..

[33] Ahern CA, Payandeh J, Bosmans F, Chanda B (2016). The hitchhiker’s guide to the voltage-gated sodium channel galaxy. J. Gen. Physiol..

[34] Feldman CR (2016). Is there more than one way to skin a newt? Convergent toxin resistance in snakes is not due to a common genetic mechanism. Heredity.

[35] Tarrant AM (2014). Transcriptional profiling of reproductive development, lipid storage and molting throughout the last juvenile stage of the marine copepod *Calanus finmarchicus*. Front. Zool..

[36] Catterall WA (2012). Voltage‐gated sodium channels at 60: structure, function and pathophysiology. Journal Physiol..

[37] Du Y, Nomura Y, Liu Z, Huang ZY, Dong K (2009). Functional expression of an arachnid sodium channel reveals residues responsible for tetrodotoxin resistance in invertebrate sodium channels. J. Biol. Chem..

[38] Kültz D (2005). Molecular and evolutionary basis of the cellular stress response. Annu. Rev. Physiol..

[39] Roncalli V, Cieslak MC, Passamaneck Y, Christie AE, Lenz PH (2015). Glutathione S-Transferase (GST) Gene Diversity in the Crustacean *Calanus finmarchicus*–Contributors to Cellular Detoxification. PloS one.

[40] Terlau H (1991). Mapping the site of block by tetrodotoxin and saxitoxin of sodium channel II. FEBS Lett..

[41] Dong K (2007). Insect sodium channels and insecticide resistance. Invert. Neurosci..

[42] Geffeney S, Brodie ED, Ruben PC (2002). Mechanisms of adaptation in a predator-prey arms race: TTX-resistant sodium channels. Science.

[43] Sorensen JS, Turnbull CA, Dearing MD (2004). A specialist herbivore (*Neotoma stephensi*) absorbs fewer plant toxins than does a generalist (*Neotoma albigula*). Physiol. Biochem. Zool..

[44] Glendinning JI (2007). How do predators cope with chemically defended foods?. Biol. Bull..

[45] Núñez-Acuña, G., Aballay, A. E., Hégaret, H., Astuya, A. P., Gallardo-Escárate, C. Transcriptional responses of *Mytilus chilensis* exposed *in vivo* to saxitoxin (STX). *J. Mollusc. Stud*. eyt030 (2013).

[46] Fabioux C (2011). Exposure to toxic *Alexandrium minutum* activates the detoxifying and antioxidant systems in gills of the oyster *Crassostrea gigas*. Harmful Algae.

[47] Gubbins MJ (2000). Paralytic shellfish poisoning toxins induce xenobiotic metabolizing enzymes in Atlantic salmon (*Salmo salar*). Mar. Environ. Res..

[48] Suzuki T, Ichimi K, Oshima Y, Kamiyama T (2003). Paralytic shellfish poisoning (PSP) toxin profiles and short-term detoxification kinetics in mussels *Mytilus galloprovincialis* fed with the toxic dinoflagellate *Alexandrium tamarense*. Harmful Algae.

[49] Dutz J (1998). *Alexandrium lusitanicum*: relationship between feeding and egg production. Mar. Ecol. Prog. Ser..

[50] Asselman J (2012). Identification of pathways, gene networks, and paralogous gene families in *Daphnia pulex* responding to exposure to the toxic cyanobacterium *Microcystis aeruginosa*. Environ. Sci. Technol..

[51] Hassett, R. P. Effects of the red-tide dinoflagellate *Alexandrium tamarense* on copepod digestion: toxin level does not predict physiological impact. *EOS Transaction*s 76, OS12G-3 (1996). cited in [Teegarden, G. J., Cembella, A. D., Capuano, C. L., Barron, S. H. and Durbin, E. G. Phycotoxin accumulation in zooplankton feeding on *Alexandrium fundyense—vector or sink?. J P*lankton Res. **25**(4), 429–443 (2003)].

[52] Hamasaki K, Takahashi T, Uye SI (2003). Accumulation of paralytic shellfish poisoning toxins in planktonic copepods during a bloom of the toxic dinoflagellate *Alexandrium tamarense* in Hiroshima Bay, western Japan. Marine Biology.

[53] Bricelj VM, Lee JH, Cembella AD, Anderson DM (1990). Uptake kinetics of paralytic shellfish toxins from the dinoflagellate *Alexandrium fundyense* in the mussel *Mytilus edulis*. Mar. Ecol. Progr. Ser..

[54] Engel P, Moran NA (2013). Thegut microbiota of insects–diversity in structure and function. FEMS Microbi. Rev..

[55] Rice P, Longden I, Bleasby A (2000). EMBOSS: The European Molecular Biology Open Software Suite. Trends Genet..

[56] Bray NL, Pimentel H, Melsted P, Pachter L (2016). Near-optimal probabilistic RNA-seq quantification. Nature Biotechnol..

[57] Robinson MD, McCarthy DJ, Smyth G (2010). K. edgeR: a Bioconductor package for differential expression analysis of digital gene  expression data. Bioinformatics.

[58] Langmead, B., Trapnell, C., Pop, M., Salzberg, S. L. Ultrafast and memory-efficient alignment of short DNA sequences to the human genome. *Genome Biol*. **10**(3) (2009). 10.1186/gb-2009-10-3-r25 PMC2690996 19261174

[59] Conesa A (2005). Blast2GO: a universal tool for annotation, visualization and analysis in functional genomics research. Bioinformatics.

