# Supplementary material for: Complementary mechanisms for neurotoxin resistance in a copepod
Source: Sci Rep. 2017 Oct 27;7:14201. doi: 10.1038/s41598-017-14545-z (PMC5660226; doi:10.1038/s41598-017-14545-z)

Supplementary material

Complementary mechanisms for neurotoxin resistance in a copepod

Vittoria Roncalli*, Petra H. Lenz, Matthew C. Cieslak and Daniel K. Hartline

Methods

Naupliar response to *A. fundyense*

Field collection and cultivation of C. finmarchicus

Copepod development consists of an embryonic stage ("eggs") followed by six naupliar (NI-NVI) and six copepodite (CI-CVI) stages. Here, the transcriptomic response to *A. fundyense* was investigated in the "late naupliar stage" consisting of a mix of NV and NVI individuals. These developmental stages were obtained by raising nauplii from eggs produced in the laboratory. *C. finmarchicus* were collected using a vertical net tow (75 cm diameter, 560 µm mesh) on July 1, 2012 in the Gulf of Maine near Mount Desert Rock (Lat: 44° 2'N; Long: 68°3'W). Adults were sorted from the plankton collection and transferred into baskets with bottoms covered with 560 µm mesh and suspended in 3.5 L culture jars of seawater containing live *Rhodomonas* sp. Eggs were collected and reared in a batch that differed in age by less than 48 hrs. After the nauplii reached the feeding stage (NIII) *Rhodomonas* sp. was added to the container. Nauplii were checked every 2-3 days, and after they reached the targeted developmental stages (NV-NVI), they were removed from culture and incubated with the experimental food as described below. All copepod cultures and experiments were maintained in a Percival Model I-36VL Incubator System (Percival Scientific, Inc., Perry, IA, USA) with temperature set at 10 ºC and the light:dark cycles set at 14L:10D.

Experimental design

For the experiment, 70-86 *C. finmarchicus* late nauplii (NV-NVI) were transferred into 100 mL crystallizing dishes with filtered seawater and fed for two days on one of two experimental diets: control and high dose of *A. fundyense* (HD) (Supplementary Material, table S2). Three biological replicates were set up per treatment (control, HD). The control and *A. fundyense* phytoplankton cultures used in this study were the same as those in three parallel studies [1-3]. Briefly, the toxic dinoflagellate *A. fundyense* (clone GTCA28, origin: western Gulf of Maine, isolated in 1985) cultures were grown at 15 °C on a 14:10 h light:dark cycle. During the experiments, the cultures were maintained at the same light:dark cycle and 10 ºC, and diluted by 50% every two days with f/2-Si medium [1-3]. The flagellate *Rhodomonas* sp. (clone CCMP739) was maintained at 15–16 °C in ambient natural light and diluted by 50% with f/2 medium every three days [1-3]. The toxicity of *A. fundyense* was measured daily during the experiment as reported in Roncalli et al., (2016) [1].

In the nauplius experiment, the non-toxic flagellate *Rhodomonas* sp. was added daily at 8,000 cells $mL^{-1}d^{-1}$ to each control replicate. Nauplii in the HD group were fed a diet of 100% *A. fundyense* at daily rations of 200 cells $mL^{-1}d^{-1}$ per experimental replicate. Estimated carbon content for the two treatments was similar with 304 and 358 µgC L^{-1} respectively for the control and HD treatments [1-3]. Nauplii were checked under a dissecting microscope to assess mortality, algal ingestion (colored/filled guts), possible malformations (none were found) and behavior (active swimming, escape swims) after 1 and 2 days. On day 2, nauplii were harvested from each treatment and biological replicate and immediately processed for RNA extraction.

RNA extraction, gene library preparation and RNA-Seq

Total RNA was extracted from pooled nauplii from each replicate (Supplementary Material, table S2) using QIAGEN RNeasy Mini Kit (QIAGEN Inc., Valencia, CA, USA), in

conjunction with a Qiashredder column (QIAGEN Inc.), following the instructions of the manufacturer, and with a final elution volume of 30 µl. Total RNA concentration and quality were checked using an Agilent Model 2100 Bioanalyzer (Agilent Technologies, Inc., Santa Clara, CA, USA). The six samples (3 biological replicates × 2 treatments) were shipped on dry ice to the University of Georgia Genomics Facility for library preparation and Illumina sequencing. There, the TruSeq RNA sample preparation kit (Illumina) was used to prepare double-stranded multiplexed cDNA libraries starting from the total RNA following manufacturer's instructions. Briefly, RNA samples were first purified with two oligo-dT selection (poly (A) enrichment using oligo-dT beds) to select for mRNAs, and then fragmented and reverse transcribed into double-stranded complementary cDNA. Each sample was tagged with an indexed adapter prior to shipping to University of Missouri DNA Core Facility (*http://biotech.missouri.edu/dnacore*) for sequencing. At the Missouri facility the samples were loaded into a single lane and sequenced on an Illumina HiSeq 2000 instrument using paired-end sequencing (100 bp). Summary of RNA Seq yields are found in Supplementary Material, table S3.

Mapping of short reads and identification of differentially expressed genes (DEGs)

Illumina sequencing for the six RNA-Seq libraries generated more than 121 million reads with 16 to 24 million reads per library with an average of 20 million across all samples (100 bp, paired-end) (Supplementary Material, table S3). Libraries were quality filtered (FASTX Toolkit, v. 0.013; http://hannonlab.cshl.edu/fastx_toolkit/) by trimming the first nine and the last 29 bases. This was followed by the elimination of low quality reads (cutoff "Phred" score = 20) as well as Illumina adapters. An average of 24% of reads were removed, leaving from 12 to 18 million reads per sample for relative gene expression analysis. Each quality filtered RNA-Seq

library was then mapped to an existing *C. finmarchicus* reference transcriptome [4,5] (96,090 contigs) using the software Bowtie [6] (v. 2.0.6). The reference transcriptome was generated through the *de novo* assembly of over 400 million reads from six developmental stages (embryo, early nauplius, late nauplius, early copepodite, late copepodite, adult female) as described previously and available online [5]. The reference transcriptome was designed to minimize ambiguous mapping (≤ 1% of mapped reads mapped > 1 time), thus, it did not include splice variants, and the $Na_V1.1$ channel was represented by a single variant. Hence, a separate analysis was used to determine relative expression of the $Na_V1.1$ variants (see text). Relative expression was calculated as reads per kilobase per million mapped reads (RPKM) for each gene by dividing the counts by the length of the transcript and the total number of mapped reads in each library using a custom script written in Perl (https://github.com/LenzLab/RNA-seq-scripts).

Differential gene expression analysis and calculation of fold-change difference in expression were performed using the BioConductor package edgeR [7]. Prior to statistical analysis, libraries were normalized as implemented by edgeR using the Trimmed mean of M values (TMM) and genes with low expression (< 1 count per million) were removed leaving 23,915 genes for statistical testing of differentially expressed genes (DEGs). The remaining genes in the reference transcriptome were either not expressed in late naupliar stages (43,470 sequences with 0 counts) [28] or expression was below the filter cutoff (1 cpm). Differentially expressed genes were identified using a pair-wise comparison between the control and experimental treatment: CONTROL vs HD. Transcripts were identified as differentially expressed using the Exact test ($p<0.05$) followed by a multiple comparison correction using the Benjamini-Hochberg method (false discovery rate <5%) as implemented by edgeR [7]. Relative expression was quantified as a ratio in units of Log_2 (experimental/control) where a value of 0 represents equal expression

between the experimental condition and control.

Functional annotation of differentially expressed genes (DEGs)

Functional annotation for genes identified as differentially expressed was undertaken using a local blast webserver. *Blastx* algorithm was used to search against the NCBI SwissProt protein database (downloaded on 18th January, 2016) onto a local Beowulf Linux computer cluster; a maximum E-value for annotation of 10^{-3} was employed. The *Blast* search yielded annotation for 66% of the DEGs. The resulting Blast annotations were then used to retrieve Gene Ontology (GO) terms with UniProt (http://www.uniprot.org/uploadlists/) under three categories: biological processes, molecular function and cellular component, which are hierarchically organized into levels. The maximum E-value used for this analysis was of 10^{-6}. Enrichment analysis was performed separately for up- and down-regulated genes with GO terms (426 and 96 respectively) against the 10,344 genes with assigned GO terms in the *C. finmarchicus* reference transcriptome [4,5]. This analysis was comparable to an earlier study on the effects of *A. fundyense* on gene expression of adult females [5]. The analysis was implemented using the software BLAST2GO (v. 2.6.4) performing the Fisher's Exact Test followed by Multiple Testing correction of False Discovery rate (FDR <5%) [8]. It is important to note that in many cases multiple functions (GO terms) are assigned to individual genes.

References

1. Roncalli, V., Turner, J.T., Kulis, D., Anderson, D.M., Lenz, P.H. The effect of the toxic dinoflagellate *Alexandrium fundyense* on the fitness of the calanoid copepod *Calanus finmarchicus*. *Harmful Algae* **51**, 56–66 (2016).
2. Roncalli, V., Cieslak, M.C., Lenz,P.H. Transcriptomic responses of the calanoid copepod *Calanus finmarchicus* to the saxitoxin producing dinoflagellate *Alexandrium fundyense*. *Scientific Reports* **6** (2016).
3. Roncalli, V., Jungbluth, M.J., Lenz, P.H. Glutathione S-transferase regulation in *Calanus finmarchicus* feeding on the toxic dinoflagellate *Alexandrium fundyense*. *PloS one*, **11**,

p.e0159563 (2016).
4. Roncalli, V., Cieslak,M.C., Lenz, P.H. Data from: Transcriptomic responses of the calanoid copepod *Calanus finmarchicus* to the saxitoxin producing dinoflagellate *Alexandrium fundyense*. Dryad Digital Repository. http://dx.doi.org/10.5061/dryad.11978 (2016).
5. Lenz, P.H., Roncalli, V., Hassett, R.P., Wu, L.S., Cieslak, M.C., Hartline, D.K., Christie, A.E. *De novo* assembly of a transcriptome for *Calanus finmarchicus* (Crustacea, Copepoda)–the dominant zooplankter of the North Atlantic Ocean. *PloS one* **9**, e88589 (2014).
6. Langmead, B., Trapnell, C., Pop, M., Salzberg, S.L. Ultrafast and memory-efficient alignment of short DNA sequences to the human genome. *Genome Biol*. **10** (2009).
7. Robinson, M.D., McCarthy, D.J., Smyth, G.K. edgeR: a Bioconductor package for differential expression analysis of digital gene expression data. *Bioinformatics* **26**, 139–140 (2010).
8. Conesa, A. Götz, S., García-Gómez, J.M., Terol, J., Talón, M., Robles, M. Blast2GO: a universal tool for annotation, visualization and analysis in functional genomics research. *Bioinformatics* **21**, 3674–3676 (2005).
9. Tarrant, A.M., Baumgartner ,M.F., Hansen, B.H., Altin, D., Nordtug, T., Olsen, A.J. Transcriptional profiling of reproductive development, lipid storage and molting throughout the last juvenile stage of the marine copepod *Calanus finmarchicus*. *Front. Zool.* **11** (2014).

Table S1. *Calanus finmarchicus* Na_V channel sequences identified by *in silico* searches of the reference transcriptome for individuals from Gulf of Maine (GOM)[5] and Norway (NOR)[9] population. For each Na_V transcript, Domain, Accession Nos. (NCBI) are listed.

ID	Domains	GOM accession #s	NOR accession #s
Na_V1.1	I	GAXK01042241-2	GBFB01079192&3
	II-IV	GAXK01152306-23	GBFB01170433-37
Na_V1.2	I	GAXK01121434-5	GBFB01098187-90
	II-IV	GAXK01186589-90	
Na_V1.3	I	GAXK01037395-8	not found
	II		not found
	III		GBFB01005556
	IV		GBFB01027397
Na_V1.Xb *	I	GAXK01036301	GBFB01017903
i	II	GAXK01012592	GBFB01192791
e	III	GAXK01114023	not found
j	III	GAXK01009404	not found
k	III	GAXK01063206	not found
f	IV	GAXK01022998	GBFB01042925

* The "NaV1.X" category is a catchall of short sequences containing P-loops, but not necessarily all from the same gene. Sequences from GOM and NOR overlap extensively and share the same P-loops, but in general differ in length and in a few corresponding residues.

Table S2. Summary of parameters monitored in *C. finmarchicus* late nauplii during 2-day experiments (day0 to day2). For both CONTROL (100% *Rhodomonas* sp.) and ALEX (100% *A. fundyense*) treatments the average of three biological replicates (r1, r2, r3) is considered. Listed number of individuals, algal toxicity (ng STX eq cell^{-1}), survival rate (%), behavior and RNA-Seq harvest (# individuals used for RNA extraction).

			Measurements			
	Day	Individuals (#)*	Algal toxicity (ng STX eq cell^{-1})	Survival (%)	Behavior**	RNA-SeqΩ
CONTROL						
	day 0	74	-	100	A	-
	day 1	74	-	100	A	-
	day2	74	-	100	A	74
ALEX	day 0	80	0.01	100	A	-
	day 1	80	0.01	95	B	-
	day2	80	0.02	95	B	75

* Average of 3 biological replicates: CONTROL (r1=74, r2=72,r3=77) and ALEX (r1=70, r2=84,r3=86).
** Behavior observation: A=active swimming, B= inactive on the bottom of container
Ω Average of 3 biological replicates: CONTROL (r1=74, r2=72,r3=77) and ALEX (r1=66, r2=80,r3=81).

Table S3. Summary of sequencing and mapping results for *C. finmarchicus* late nauplii (NV-NVI) feeding on CONTROL (100% *Rhodomonas* sp.) and ALEX (100% *A. fundyense*) for 2 days. Each treatment consists of 3 biological replicates (r1, r2, r3). For each replicate number of Illumina sequenced reads (100bp), number of high-quality filtered reads used for the mapping, mapping overall alignment rate (%) and number of reads that mapped 1 time.

Samples	Raw reads (#)	Reads for mapping (#)*	Overall alignment (%)	Mapped reads 1 time (#)
CONTROL				
r1	18,838,318	14,192,172	73	10,253,391
r2	19,212,698	14,381,198	73	10,378,663
r3	24,038,802	18,106,969	73	13,072,043
ALEX				
r1	20,478,974	15,531,901	72	11,095,580
r2	16,820,948	12,759,955	73	9,178,538
r3	22,361,678	15,271,110	74	11,061,185

* Raw reads were quality filtered (cutoff "Phred" score = 20 and removal of Illumina adapters) and trimmed (first nine and the last 29 bases) prior to mapping

Table S4. Gene Ontology (GO) enrichment analysis. List of enriched GO terms for up-and down-regulated genes in *C. finmarchicus* late nauplii (NV-NVI) feeding on high dose (HD) treatment of *A. fundyense* for 2 days.

	Term description	GO	Category	P-Value
Up-regulated				
	Transport	GO:0006810	BP	P=0.018
	Establishment of localization	GO:0051234	BP	P=0.018
	Localization	GO:0051179	BP	P=0.018
	Developmental process	GO:0032502	BP	P=0.013
	Cell	GO:0005623	CC	P=0.024
	Cytoplasmic part	GO:0044444	CC	P=0.026
	Extracellular region	GO:0005576	CC	P=0.033
	Cell part	GO:0044464	CC	P=0.036
	Intracellular organelle part	GO:0044446	CC	P=0.048
	Organelle part	GO:0044422	CC	P=0.048
Down-regulated				
	Cellular amino acid metabolic process	GO:0006519	BP	P=0.0003
Gene ontology term (GO) Category: (BP) Biological process, (MF) Molecular function (MF) and (CC) Cellular component.				

Table S5. Comparison of differentially expressed genes (DEGs) and fold change expression between late nauplii and adult femlaes feeding of *A. fundyense* (HD) for 2 days. Genes were identified as differentially expressed using the Exact test (P<0.05) and a multiple correction using Benjamini-Hochberg method (false discovery rate <5%) as implemented by edgeR [7]. Data for adult females are publicly available [4].

	Late nauplii	Adult females
Total DEGs (#)	814*	1,388
Up-regulated (#)	622	943
Fold change 2-4 (%)	90	54
Down-regulated (#)	192	445
Fold change 2-4 (%)	94	91
* Statistical significant difference: X^2 test=66.02; p<0.000001		

Table S6. List of selected genes involved in protein turnover differentially expressed in *C. finmarchicus* late nauplii and adult females feeding on *A. fundyense* for 2 days. Genes are classified in different protein families based on their blast annotation. For each gene Accession No. (NCBI) and relative fold change in expression (absolute) are listed. The direction of expression (up- or down-regulated) and the magnitude are indicated by arrows ("red = up" and "green = down" regulated genes). Relative expression for all the serine proteases was similar in the two stages and ranged between 1 and 52 RPKM.

**Protein family**	**Nauplii**	**A. females**
Serine protease 3		
GAXK01018947	↑	
GAXK01018996	↑	
GAXK01127960	↑↑	
GAXK01163745	↑	
GAXK01188611		↑
GAXK01076666		↑
GAXK01116212		↑↑
GAXK01081763	↑	↑↑↑
Serine protease 6		
GAXK01031646		↑↑↑
GAXK01011395		↓↓
Serine protease 9		
GAXK01110169	↑	
GAXK01027116	↑	
GAXK01099048	↑	
Serine protease 14		
GAXK01069221		↑↑
GAXK01098506		↑↑
GAXK01136255		↑↑
Serine protease easter		
GAXK01105326		↑
GAXK01188932		↑
GAXK01097615		↑
Serine proteinase stubble		
GAXK01020344		↑
GAXK01019334		↑
GAXK01011395		↑

**Up-regulated**	**Down-regulated**
↑ <2.5 fold change	↓ <2.5 fold change
↑↑ 2.5-3.9 fold change	↓↓ 2.5-3.9 fold change
↑↑↑ > 4 fold change	↓↓↓ > 4 fold change

Figure S1. Magnitude of response. Fold change of differentially expressed genes (DEGs) in *C. finmarchicus* late nauplii feeding on *A. fundyense* (HD) for 2 days. A) up-regulated genes and B) down-regulated genes. Fold change is absolute.

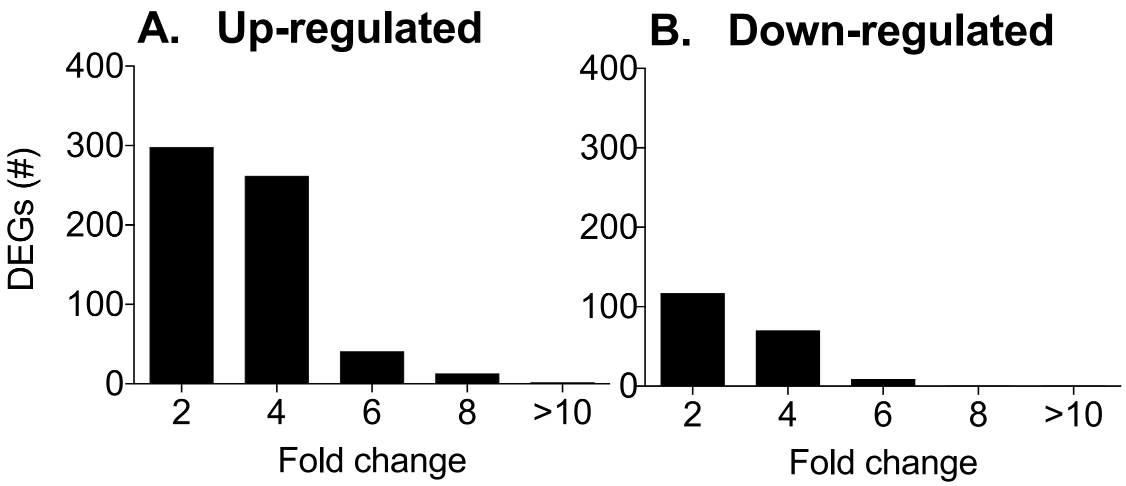

Figure S2. Biological processes represented in the nauplius response to *A. fundyense*. Pie chart of the annotated DEGs regulated in *C. finmarchicus* late nauplii feeding on *A. fundyense* HD diet for 2 days. The pie chart includes Gene Ontology (GO) terms belonging to the biological process (BP) category.

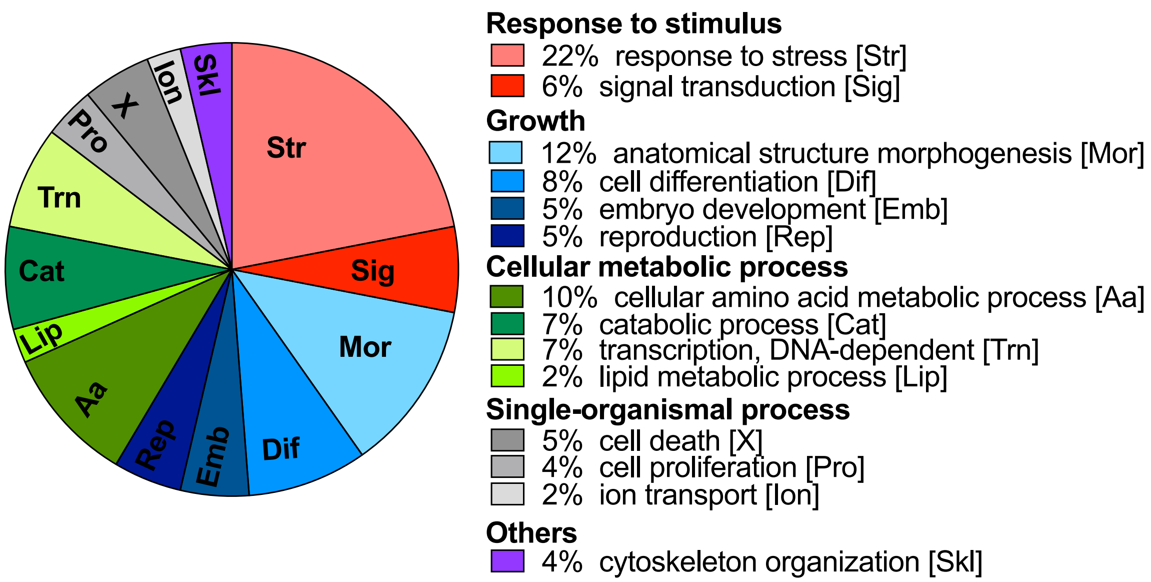

Supplement: Supplementary file 1 — Supplementary material [file 41598_2017_14545_MOESM1_ESM.pdf]
